# Deformation of two-phase aggregates with *in situ* X-ray tomography in rotating Paris–Edinburgh cell at GPa pressures and high temperature

**DOI:** 10.1107/S1600577523005374

**Published:** 2023-07-19

**Authors:** Tommaso Mandolini, Julien Chantel, Sébastien Merkel, Yann Le Godec, Nicolas Guignot, Andrew King, Jerome Hosdez, Laura Henry, Nadège Hilairet

**Affiliations:** a Univ. Lille, CNRS, INRAE, Centrale Lille, UMR 8207 – UMET – Unité Matériaux et Transformations, F-59000 Lille, France; b Institut de Minéralogie, de Physique des Matériaux et de Cosmochimie (IMPMC), UMR CNRS 7590, Muséum National d’Histoire Naturelle, IRD UMR 206, Sorbonne University, 75005 Paris, France; c Synchrotron Soleil, L’Orme des Merisiers, 91192 Saint-Aubin, France; d CNRS, Centrale Lille, UMR 9013 – LaMcube – Laboratoire de Mécanique, Multiphysique, Multiéchelle, University of Lille, 59000 Lille, France; University of Malaga, Spain

**Keywords:** high pressure, *in situ* X-ray tomography, deformation, two-phase aggregates

## Abstract

High-pressure (>1 GPa) torsion apparatus can be coupled with *in situ* X-ray tomography (XRT) to study microstructures in materials associated with large shear strains. Here, deformation experiments were carried out on multi-phase aggregates at ∼3–5 GPa and ∼300–500°C, using a rotational tomography Paris–Edinburgh press (RoToPEc) with *in situ* absorption contrast XRT on the PSICHE beamline at Synchrotron SOLEIL. The actual shear strain reached in the samples was quantified with respect to the anvil twisting angles, which is γ ≤ 1 at 90° anvil twist and reaches γ ≃ 5 at 225° anvil twist. 2D and 3D quantifications based on XRT that can be used to study *in situ* the deformation microfabrics of two-phase aggregates at high shear strain are explored. The current limitations for investigation in real time of deformation microstructures using coupled synchrotron XRT with the RoToPEc are outlined.

## Abstract 2

The use of a high-pressure torsion apparatus to deform multi-phase aggregates under high shear strain is explored, and *in situ* X-ray tomography data at high pressure and high temperature are collected. Step-by-step procedures on strain measurements and image processing are outlined, and results on the studied materials are presented in 2D and 3D.

## 1. Introduction

Rock deformation is one of the key processes that control the dynamics in the Earth. Rocks are by nature heterogeneous materials that comprise multiple phases, which are collectively involved in the deformation of the rock and determine its bulk deformation behaviour (*e.g.* White *et al.*, 1980 ▸). Because of the complexity of rock deformation, the microstructure can be best characterized in 3D (*e.g.* Bryon *et al.*, 1995 ▸; Mock & Jerram, 2005 ▸; Holzer *et al.*, 2006 ▸; Jerram & Higgins, 2007 ▸; Fusseis *et al.*, 2014*a* ▸; Morales *et al.*, 2018 ▸).

X-ray tomography (XRT) is a non-destructive technique allowing 3D investigation of a large range of materials (*e.g.* Denison & Carlson, 1997*a* ▸,*b* ▸; Van Geet *et al.*, 2001 ▸; Koeberl *et al.*, 2002 ▸; Tarplee *et al.*, 2011 ▸; Fusseis *et al.*, 2014*b* ▸). Deformation experiments with *in situ* XRT permit the microstructures at different deformation stages to be recovered, removing part of the ambiguities in their interpretation and the need to carry out fastidious series of experiments at different strains. *In situ* XRT coupled with experimental deformation devices is now possible in laboratories (*e.g.* McBeck *et al.*, 2020 ▸; Shi *et al.*, 2020 ▸, 2021 ▸) and in synchrotron facilities (*e.g.* Wang *et al.*, 2011 ▸; Kareh *et al.*, 2012 ▸; Fusseis *et al.*, 2012 ▸, 2014*a* ▸; Philippe *et al.*, 2016 ▸; Butler *et al.*, 2017 ▸; Colombier *et al.*, 2020 ▸; Dobson *et al.*, 2020 ▸; Marone *et al.*, 2020 ▸; Guignot *et al.*, 2020 ▸).

Torsion apparatus are very relevant for earth science materials because they allow studying microstructures associated with large geological shear strains (*e.g.* Paterson & Olgaard, 2000 ▸). Torsion set-ups with *in situ* XRT exist at ambient or ‘low’ pressures (≤0.1 GPa) (Fusseis *et al.*, 2014*a* ▸; Renard *et al.*, 2016 ▸; Di Genova *et al.*, 2016 ▸; Voltolini *et al.*, 2019 ▸; Dobson *et al.*, 2020 ▸) and high pressures (>1 GPa) (*e.g.* Yamazaki & Karato, 2001 ▸; Wang *et al.*, 2005 ▸, 2011 ▸; Le Godec *et al.*, 2009 ▸, 2012 ▸; Yu *et al.*, 2016 ▸; Philippe *et al.*, 2016 ▸). The highest pressures (>20 GPa) and temperatures (>2000°C) were reported in a torsion device called the rotational Drickamer apparatus (*e.g.* Girard *et al.*, 2016 ▸; Yamazaki & Karato, 2001 ▸). The increase in mean stress (pressure) and temperatures deep within the Earth and rocky planets modifies deformation mechanisms, hence the need to investigate rocks using such high-pressure apparatus.

High-pressure torsion apparatus with *in situ* XRT only exist at a few synchrotron beamlines (*e.g.* Wang *et al.*, 2005 ▸, 2011 ▸; Álvarez-Murga *et al.*, 2017 ▸; Philippe *et al.*, 2016 ▸). One is a Drickamer cell-based module (*e.g.* Wang *et al.*, 2005 ▸, 2011 ▸) at the GeoSoilEnviro-CARS (GSECARS) beamline at the Advanced Photon Source (APS) (IL, USA). Another is the rotating tomography Paris–Edinburgh cell (RoToPEc) (Philippe *et al.*, 2016 ▸), which has been in use at the PSICHE beamline (*e.g.* King *et al.*, 2016 ▸, 2019 ▸) at Synchrotron SOLEIL (Saint-Aubin, France), on the ID27 beamline at the ESRF (Grenoble, France) (Álvarez-Murga *et al.*, 2017 ▸; Chakraborti *et al.*, 2022 ▸) and I11 beamline at Diamond Light Source synchrotron (Oxfordshire, UK) (*e.g.* Philippe *et al.*, 2016 ▸). The use of these apparatus for deformation studies with *in situ* XRT is scarcely documented.

Here, we report the use of the RoToPEc, a modified design of the V7-type Paris–Edinburgh cell (PEc) (*e.g.* Le Godec *et al.*, 2009 ▸) with a rotating module inspired by the cell by Bromiley *et al.* (2009 ▸). The RoToPEc can allow pressures up to ∼10 GPa and temperatures up to ∼2000°C (Philippe *et al.*, 2016 ▸). Philippe *et al.* (2016 ▸) and Álvarez-Murga *et al.* (2017 ▸) suggested its potential to image samples *in situ* at simple shear strain of γ > 2 or γ > 3. It has been employed for studying metallic phase transitions (Boulard *et al.*, 2020 ▸) and melt percolation (Berg *et al.*, 2018 ▸) at high pressure and temperature, but without the use of controlled deformation.

In this work, we perform deformation with *in situ* absorption contrast XRT in the RoToPEc on two different mineralogical aggregates relevant for earth science: olivine + serpentine, and pyroxene + garnet (see Section 2.3). These aggregates are common in the Earth lithosphere, and can be representative for large geologically shear strain environments that can be experimentally explored through torsion. The experiments were performed at high pressure (HP; ∼3–5 GPa) and temperature (HT; ∼300–500°C), and conducted on the PSICHE beamline at Synchrotron SOLEIL. From a hardware point of view, we provide a temperature calibration for the cell, and report pressures and strains with increasing anvil twist in the cell/samples. From the point of view of microstructural data, we report our analysis workflow, processing possibilities and their limitations. With these, we illustrate potential for microstructural observations and analysis in our tomographic datasets, providing representative examples of 2D and 3D quantifications with increasing deformation. We also outline possible improvements for the instruments and methodology all along the different sections.

## 2. Experimental procedure

### 2.1. High-pressure apparatus and cell assembly

**Figure 1:**
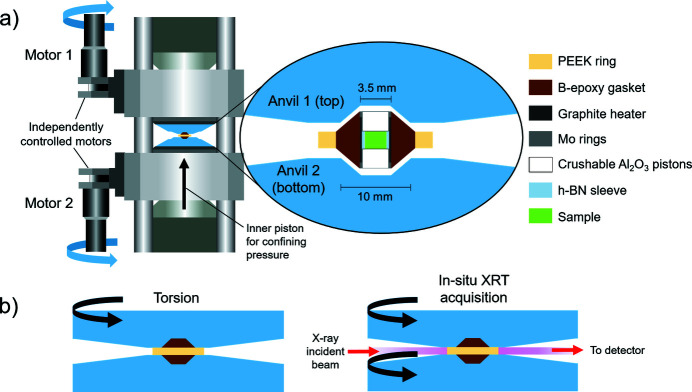
(*a*) RoToPEc and assembly designs for hot-pressing and deformation. (*b*) Types of anvil motions for torsion (left) and acquisition of *in situ* XRT (right). Anvil motions are controlled by the motors in (*a*): motor 1 for torsion; motor 1+2 for *in situ* XRT acquisition.

Fig. 1 ▸ shows the RoToPEc and assembly employed for hot-pressing the starting materials (see Section 2.3) and subsequent deformation (torsion). Two opposed anvils squeeze the sample under the uniaxial load, which is transmitted through a hydraulic piston located in the lower press frame [Fig. 1 ▸(*a*)]. Under uniaxial load, two individually controlled motors [Fig. 1 ▸(*a*)] can permit two types of anvils motions [Fig. 1 ▸(*b*)] (*e.g.* Philippe *et al.*, 2016 ▸): (i) an anvil rotates while another remains fixed in the press frame for inducing torsion (or twist) to the sample; (ii) both anvils rotate in the same direction for *in situ* XRT collection. It is not possible to collect *in situ* XRT while inducing torsion, since it has to be stopped before initiating the simultaneous anvils rotation.

In this work, a 10/3.5 mm assembly was employed [Fig. 1 ▸(*a*)]. The sample was surrounded by a h-BN (hexagonal boron nitride) sleeve, and in contact with crushable alumina pistons on top and bottom [Fig. 1 ▸(*a*)]. A boron/ep­oxy mixture (5/1), almost transparent to X-rays, was used to make the gasket (or pressure-transmitting medium), which was surrounded by a PEEK ring [Fig. 1 ▸(*a*)]. This served to contain the sideways expansion of the assembly during the experiments.

### 2.2. Temperature in the cell

To our knowledge, temperature calibrations have been reported for a 7/3.5 mm assembly and for a Paris–Edinburgh-type cell (*e.g.* Riva *et al.*, 2018 ▸; Y. Le Godec, personal communication), relying on DC (direct current) power. These calibrations cannot directly be used in the present study because of the difference in apparatus, assembly design and size, as well as power, since the PSICHE beamline uses AC (alternative current). Therefore, here we document our offline calibration for the 10/3.5 mm assembly in the RoToPEc to use at PSICHE with AC power supply.

The calibration was performed using a thermocouple (type D), up to 700°C. The apparatus loads were 42.5 tons and 25.5 tons, corresponding to hydro­static pressures in the cell of ∼4 GPa and ∼2 GPa, respectively. Hydro­static pressure is estimated from a beam-time session experiment, using the same assembly, during which we collected *in situ* energy-dispersive (white-beam mode) X-ray diffraction from the h-BN sleeve [Fig. 1 ▸(*a*)]. The h-BN 002 diffraction line and the *c*-axis compressibility were used to calculate the pressure (Le Godec *et al.*, 2000 ▸).

**Figure 2:**
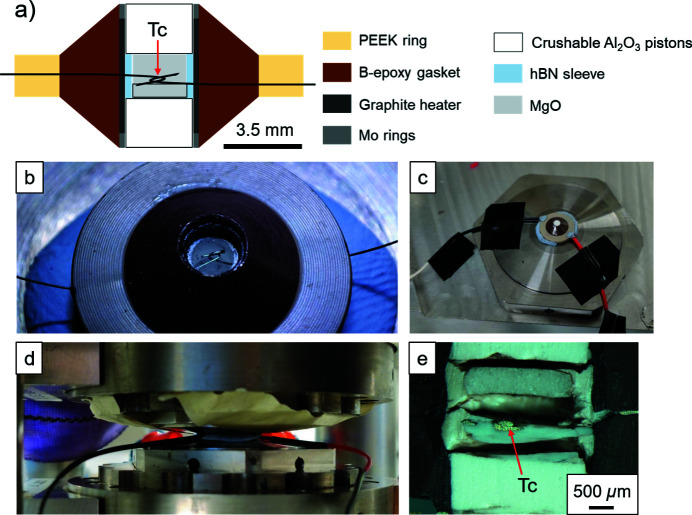
Assembly for temperature-calibration experiments in the RoToPEc. (*a*) Assembly with thermocouple. Tc indicates the position of the thermocouple junction. (*b*) Detail of the thermocouple junction. (*c*) Assembly on one anvil before loading. Sticky tack (light blue) surrounds the PEEK, and covers the holes. (*d*) Assembly under pressure between the anvils. (*e*) Recovered assembly showing the thermocouple junction (Tc) being close to the centre of the assembly.

The thermocouple was placed in the equatorial plane of the gasket and at the centre of the cell assembly (Fig. 2 ▸). The extremities of the thermocouple wires were bent to make a junction [Figs. 2 ▸(*a*) and 2(*b*)]. A cold-compressed MgO (magnesium oxide) plug was placed below the junction. A compacted fine-grained powder of MgO was used to surround the thermocouple junction [Fig. 2 ▸(*a*)]. To keep the PEEK ring in place and prevent it from breaking during pressurization, sticky tack was placed on two opposite sides of the PEEK ring [Fig. 2 ▸(*c*)].

**Figure 3:**
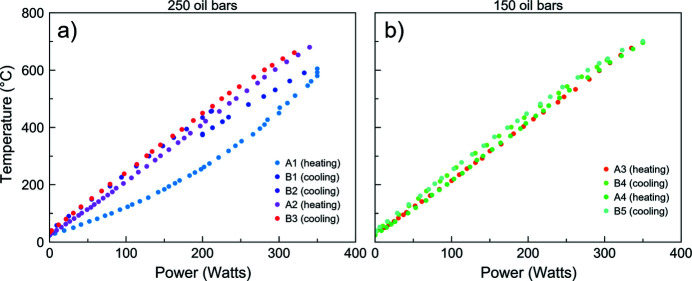
Power versus temperature collected in the same run at (*a*) 250 bar (∼4 GPa) and (*b*) 150 bar (∼2 GPa).

Power (W) versus temperature (°C) data were collected during a single run, at two different loads: first at 42.5 tons (∼4 GPa) and then at 25.5 tons (∼2 GPa) (Fig. 3 ▸).

At higher pressure, the first cycle of heating and cooling [A1 and B1; Fig. 3 ▸(*a*)] shows a different trend compared with the following ones [from B2 to B3, Fig. 3 ▸(*a*)], with a heater average resistance of 40 mΩ. The change in slope in A1 [Fig. 3 ▸(*a*)] could indicate a change of heater efficiency: below ∼100 W, the slope is much lower. The heater achieves the best efficiency after the cycle B2, where trends stabilize and the average resistance is 44 mΩ at 250 bar. The average resistance is 48 mΩ at 150 bar. Such variation in efficiency may be due to the impurities in the graphite heater, or to the quality of contact with the electrodes. For this reason, trends prior to B2 were not considered for the calibration. The trends A2 and B3 at 42.5 tons (∼4 GPa) [Fig. 3 ▸(*a*)] and the ones from A3 to B5 at 25.5 tons (∼2 GPa) [Fig. 3 ▸(*b*)] were fitted using a linear relation between the temperature (°C) and the power (W). Table 1 ▸ shows the linear fit parameters for each of the two pressures. The highest power of 350 W corresponds to a temperature of 723.40 ± 14.65°C at ∼4 GPa (42.5 tons), and 719.62 ± 14.58°C at ∼2 GPa (25.5 tons).

Cooking the cell assembly in a laboratory furnace at least above ∼600°C before experiments would likely allow the effect seen here to be avoided [Fig. 3 ▸(*a*)]. Furthermore, future work could test thermal insulator materials such as zirconia to replace the alumina pistons [see Raterron *et al.* (2013 ▸) for thermal gradient when using alumina pistons in a uniaxial cell for HP deformation]. To maintain sufficient height for XRT, the pressure-transmitting medium could also be partially replaced by zirconia (*e.g.* Kono *et al.*, 2014 ▸; Riva *et al.*, 2018 ▸), keeping an equatorial window that would allow X-rays to go through.

### 2.3. Deformation experiments

The deformed aggregates are made of two or more minerals, whose chemical and density differences result in a significant X-ray absorption contrast. Data were collected for:

(i) Powders with controlled volume fraction of olivine [(Mg,Fe)_2_SiO_4_; density: ∼3.2 g cm^−3^] and serpentine [ideal formula: Mg_3_Si_2_O_5_(OH)_4_; density: ∼2.7–2.9 g cm^−3^].

(ii) Aggregates of pyroxene [(Ca,Na)(Mg,Fe)Si_2_O_6_; density: ∼3.3 g cm^−3^] and garnet [(Mg,Fe,Mn,Ca)_3_(Al,Fe)_2_(SiO_4_)_3_; density: ∼3.6 g cm^−3^], which were either retrieved from a rock specimen (M. P. Terry, personal communication; Robinson *et al.*, 2003 ▸) and ground to a powder with controlled volume fraction, or core-drilled out of another rock specimen (Locatelli *et al.*, 2018 ▸).

A list of the experiments is given in Table 2 ▸. Hot-pressing and deformation of the samples were conducted on the PSICHE beamline. A description of the beamline specifics can be found in, for example, King *et al.* (2016 ▸, 2019 ▸). Additional experiments (‘*Ex situ*’, see Table 2 ▸) were performed at the IMPMC laboratory (Sorbonne University).

We conducted the runs at pressure–temperature conditions within the stability field of the minerals in the aggregates. For the olivine + serpentine aggregates, we aimed for a confining pressure of ∼4 GPa, and temperatures were ∼300–400°C. For the pyroxene + garnet aggregates, temperatures were ∼400–500°C, and we aimed for a confining pressure of ∼3 GPa, for powders retrieved from specimen by Locatelli *et al.* (2018 ▸), and for a confining pressure of ∼4–5 GPa, for the core-drilled sample retrieved from specimen [by M. P. Terry (personal communication), Robinson *et al.* (2003 ▸)]. All these pressure and temperature conditions were chosen in order to avoid chemical reaction that would add complexity to the observations and mechanical behaviour of the aggregates.

Note that during the temperature calibration (Fig. 3 ▸) the heater reached a stable temperature response with respect to power at the second heating cycle (Section 2.2) only. Therefore, the first deformation step and tomography measurements may have been conducted at lower temperature [following the first heating cycle, Fig. 3 ▸(*a*)] than the next steps. The temperature reported in Table 2 ▸ is the one after the first cycle.

The confining pressure was determined by *in situ* energy-dispersive (white-beam mode) X-ray diffraction recorded on the h-BN, using the 002 diffraction line and the *c*-axis compressibility (Le Godec *et al.*, 2000 ▸). The torsion [Fig. 1 ▸(*b*)] was generated by rotating the top anvil at a speed of 0.02° s^−1^.

### 2.4. XRT acquisition and reconstruction

Pink-beam illumination at the PSICHE beamline (*e.g.* King *et al.*, 2016 ▸, 2019 ▸) was used to collect the tomographic datasets. The beam was filtered with a mirror to cut the high energies, then aluminium and tin filters were used to define the spectrum. This gave an average X-ray beam energy of 39 keV, with a beam size of ∼2.6 mm × 2.6 mm. With this setting, a voxel edge was 1.3 µm in the later reconstructed tomographic datasets. A speed of anvil rotation of 0.15° s^−1^ was used to collect ∼3000 projections from 0° to 180° rotation. A standard flat-field correction is applied to each acquired X-ray image. The image is corrected for inhomogeneous illumination using a white-field reference image (the beam without the sample) taken prior to each tomography scan and from the electronic noise by a dark-current image (image taken without beam).

*In situ* XRT acquisition [Fig. 1 ▸(*b*)] was performed at specific anvil twisting angles to sequentially image the deformation microstructure. In the first series of experiments (#15 to #21, excluding 15b and 18b, Table 2 ▸), the majority of the tomographic datasets were acquired at high temperature. This enhanced motions in the samples during *in situ* XRT acquisition. For instance, in #15, #19 and #21, heterogeneous motions were significant, and the tomographic datasets could not be fully corrected during pre-processing (before volume reconstruction). Therefore, in the second series of experiments (#23 to #25, Table 2 ▸), we opted for quenching the samples by shutting down the power after each twisting step, and letting them stabilize for ∼30–40 min before tomographic acquisition. Then, the samples were again heated up to the target temperature and deformed to higher twisting angles. For each deformation experiment, the twist interval was either 45° or 90°.

The maximum twisting angle depended on the gap remaining between the opposed anvils. Because of the sideways expansion of the cell assembly, this gap shortens during the experiment (by a maximum of ∼900 µm, see Section 4). Since the anvils used here are not transparent to X-rays, it constrains the tomography view, which is therefore smaller than the actual sample height (see Section 3).

**Figure 4:**
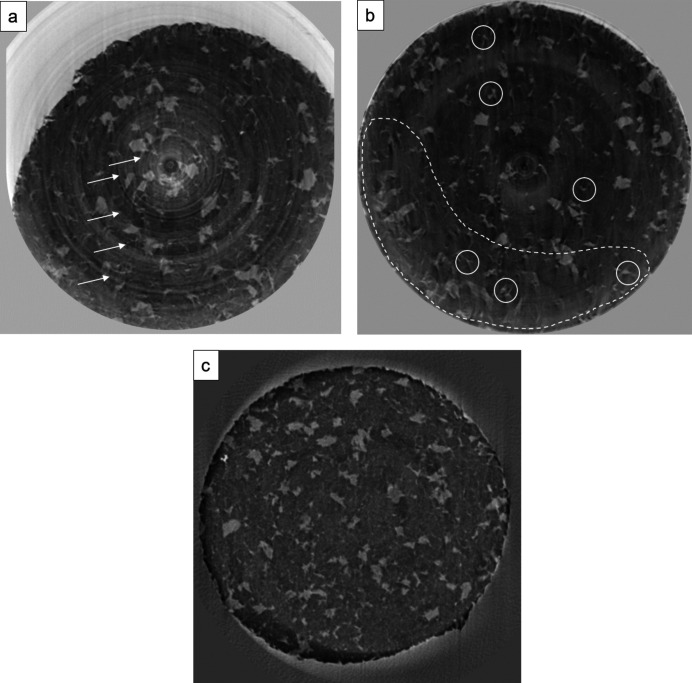
Comparison between non-satisfactory (*a*,*b*) and satisfactory (*c*) reconstructions of tomographic datasets (negative contrast) of samples of olivine + serpentine (serpentine is brighter; olivine is darker). (*a*) The position of the axis of rotation is off. White arrows show ring artefacts. (*b*) Representative example of non-satisfactory body movement correction: sample motions are still visible within the white-dotted-line area; white solid circles show ‘triple point’ structures (see text). (*c*) No visible artefacts.

XRT data were pre-processed and reconstructed at the PSICHE beamline using Python scripts (*Tomodata*) and *PyHST2* (King *et al.*, 2016 ▸; Mirone *et al.*, 2014 ▸). *PyHST2* is available at https://ftp.esrf.fr/scisoft/PYHST2/installation.html. During pre-processing of the tomographic datasets, sample motion artefacts (*e.g.* Kastner & Heinzl, 2018 ▸) were present due to the sample response to the applied deformation. Fig. 4 ▸ shows representative examples of non-satisfactory tomographic datasets due to sample motions [Figs. 4 ▸(*a*) and 4(*b*)], in comparison with a satisfactory reconstructed image [Fig. 4 ▸(*c*)].

In some cases, motions at the sample scale resulted in the position of the rotation axis being completely off [Fig. 4 ▸(*a*)], and it was necessary to repeat the acquisition. The common artefacts due to motions consisted of ring artefacts (*e.g.* Koeberl *et al.*, 2002 ▸; Wang *et al.*, 2011 ▸; Gharbi *&* Blunt, 2012 ▸; Mirone *et al.*, 2014 ▸; Berg *et al.*, 2018 ▸; Kastner & Heinzl, 2018 ▸), image ‘blurriness’, shading and ‘triple-point’ (or the so-called ‘Mercedes’) structures [Fig. 4 ▸(*b*)]. A Paganin filter (*e.g.* Paganin *et al.*, 2002 ▸) was used to reduce the rings artefacts. For the remaining artefacts, we used the so-called ‘Mercedes’ correction filter, which is implemented in the scripts at the PSICHE beamline (A. King, personal communication). The filter relies on the selection of multiple ‘triple-point’ (or ‘Mercedes’) structures, and uses them as markers from which to derive an average rigid-body movement of the sample, which is taken into account in the reconstruction by *PyHST2*. However, in some cases of significant deformation of the samples during the scan, the rigid-body description was inadequate and could not correct the whole volume [Fig. 4 ▸(*b*)]. This could be attributed to the heterogeneous nature of the samples, in particular to the distribution of the ‘weaker’ (more susceptible to deform) phase; in areas where this phase would be more frequent, the extent of the sample motion could be higher than the surroundings [Fig. 4 ▸(*b*)]. Any remaining artefacts at this stage were corrected during post-processing treatment (after volume reconstruction, see Section 5).

Finally, after applying the necessary corrections to reduce as much as possible the artefacts during pre-processing, the reconstruction using *PyHST2* was launched. The final reconstructed slices were later stacked to render 3D images in *Avizo* software (see Section 5).

## 3. Strain measurements strategy

The anvil twisting angles (Table 2 ▸) in HP torsion apparatus do not correspond to the actual strains in the samples, because deformation can be partly taken up by the cell pistons or lost in frictions at the interfaces. The transfer of strain to the samples with increasing deformation in the RoToPEc was lacking a clear systematic quantification, to our knowledge.

To measure the simple shear strain transferred to the samples in torsion experiments, one or more strain marker(s) are usually employed, as reported in previous work using the Drickamer cell-based module (*e.g.* Wang *et al.*, 2011 ▸; Girard *et al.*, 2016 ▸). The relative displacement or motion of the marker(s) can give an angle between the top and bottom surfaces of the sample, in a plane parallel to the shear direction. Then, the relation to obtain the simple shear strain γ can be 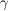 = 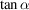 (*e.g.* Fossen, 2012 ▸), where α is the angle shown by the strain marker(s).

Here, we performed shear strain measurements in powder samples hot-pressed in the RoToPEc (experiments #23–25, Table 2 ▸) just prior to deformation. Inserting a metal foil marker (*e.g.* Girard *et al.*, 2016 ▸) within the powder was not a reliable method: simply stacking the assembly with a free metal foil inside risks unconstrained movements of the reference metal foil. Hence, we used the *in situ* XRT to identify specific particles visible at all deformation stages within the aggregates, track their motion at each anvil twisting angle, and estimate the simple shear strain.

A number of hemo-ilmenite (Fe- and Ti-rich oxides) crystals (∼10 µm) with a higher density and more absorbing elements than the other minerals (olivine and serpentine, which are mostly Mg-,Si-rich oxides) were added as markers. This mineral does not react with the sample under the experimental conditions. These particles were placed in multiple locations within the powders, *i.e.* close to either the centre or the rim of the sample. Because of the concave anvil’s geometry [Fig. 1 ▸(*b*)] and anvils gap reduction during the experiment, the anvil’s shadows partially cover the sample along its height. Hence, particles selected for strain measurements need to be close to the middle (along the height) of the sample, in the portion which remains visible for the entire experimental run. The reconstructed XRT images were used to locate at least one particle marker that should be visible for the whole experiment.

### 3.1. Total shear strain and uniaxial strain

In opposed-anvils torsion devices, such as the RoToPEc or Drickamer-based cells, the strain includes both a simple shear and a uniaxial shortening/lateral extrusion components. The uniaxial component should be reduced as much as possible, but it is unavoidable due to the geometry used to generate pressure. Both components should be measured whenever possible.

**Figure 5:**
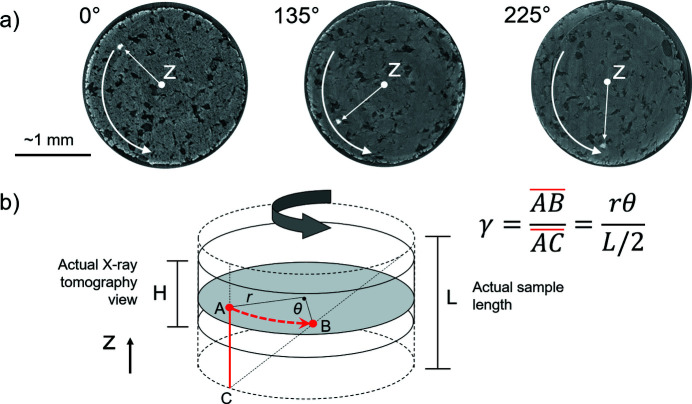
Methods for shear strain measurements. *Z* is the torsion axis. (*a*) Motion of the marker (white arrow) with increasing anvil twisting angle in XRT images. (*b*) Theoretical transfer of simple shear strain in a cylindrical sample under torsion, and formula for simple shear strain used here; *r* is the radius to the marker location, θ is the measured twisting angle in the sample, *AB* shows the marker motion and defines the arc length of θ, *AC* is half of the height of the sample, and γ is the simple shear strain.

An overview of our methodology used to calculate the strain is given in Fig. 5 ▸. The shear strain γ is calculated by measuring the motion of the marker in the plane where the transport of matter lies, perpendicular to the torsion axis [Fig. 5 ▸(*a*)]. The motion of the marker gives the real twisting angle θ transferred to the sample. Then, the relation for γ is

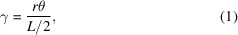

with θ expressed in radians, *r* being the radius from the centre of the sample to the location of the marker, and *L* being the measured height of the recovered sample after deformation [Fig. 5 ▸(*b*)]. The marker is located close to the centre of the sample along *L* [Fig. 5 ▸(*b*)]. The product *r*θ gives the arc length defining the marker motion [Fig. 5 ▸(*b*)]. *r* is taken as an average since the marker tends to move away from the centre of the sample as the twisting increases, possibly due to the lateral extrusion.

The lateral extrusion results in a uniaxial strain component ɛ transferred to the sample, and it is calculated via the relation

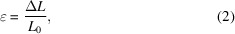

with Δ*L* being the difference between the final (after deformation) and initial (before deformation) lengths of the sample. This initial sample length *L*_0_ was measured on a reference sample recovered from a static (*i.e.* no twisting) *ex situ* experiment (#18b, Table 2 ▸) performed on the RoToPEc. The length *L*_0_ is used as a representative initial length of reference to estimate ɛ in deformed samples.

### 3.2. Total strain rates and strains at each twisting step

In the case of *in situ* experiments (at the beamline), the twisting needs to be stopped to acquire the X-ray tomography at different twisting steps [Fig. 1 ▸(*b*)]. Therefore, obtaining the actual duration of the deformation in this case is not straightforward. Alternatively, *ex situ* deformation can be carried out continuously, and the actual duration of the deformation during twisting is known. We performed an *ex situ* deformation experiment on the RoToPEc (#15b, Table 2 ▸) at the same speed of anvil rotation as for *in situ* experiments (*i.e.* 0.02° s^−1^). Hence, the total simple shear strain rate 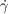 for the γ component, and the uniaxial strain rate 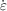 for the ɛ component, can be estimated,

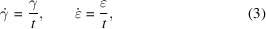

with *t* being the total duration of deformation applied in the *ex situ* experiment (∼3 h for a total of 225° anvil twisting angle).

Then, using 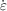 , and Δ*t*, the time interval to a specific twisting angle, the actual length *L*′ and the uniaxial strain ɛ′ can be estimated for the sample at a specific twist step,

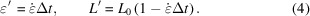

The value of *L*′ and measured angle θ′ [real twisting angle at each twist step, Fig. 5 ▸(*a*)] can be used to calculate the simple shear strain γ′ at each anvil twisting step with equation (1).

Note that this methodology to obtain simple shear and uniaxial strains at each twisting step assumes a constant uniaxial compression rate and constant shear strain rate.

### 3.3. Equivalent strain rates

Since both components of the uniaxial and simple shear are present in our samples, the equivalent strain ɛ_E_ and the equivalent strain rate 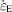 are calculated following the relations

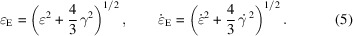




### 3.4. Summary

Table 3 ▸ shows the results from the strain measurements. The measurements refer to strain markers located close to the edge of the samples [Fig. 5 ▸(*a*)]. In one run, we could also track a marker closer to the centre of the sample, which gave lower γ and ɛ_E_ than for the marker close to the edge (#24, Table 3 ▸). This is consistent with the expected strain gradient along the radius of the samples. The highest value of simple shear strain γ transferred to the sample close to the edge is ∼5, at a strain rate 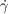 of 10^−4^ s^−1^ and a real twisting angle θ of ∼100° (225°anvil twisting angle). The highest uniaxial strain ɛ is ∼0.5 at a strain rate 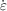 of 10^−5^ s^−1^. The highest equivalent strain ɛ_E_ is ∼600% for a 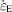 of 10^−4^ s^−1^.

## 4. Deformation effects on the assembly and sample

The sideways extrusion of the assembly/sample is one of the major difficulties in PEc-type presses during experiments, with the gap between the anvils [H, Fig. 5 ▸(*b*)] shortening. When twisting is performed in addition to compression, the extrusion is expected to be more pronounced than for a static experiment.

**Figure 6:**
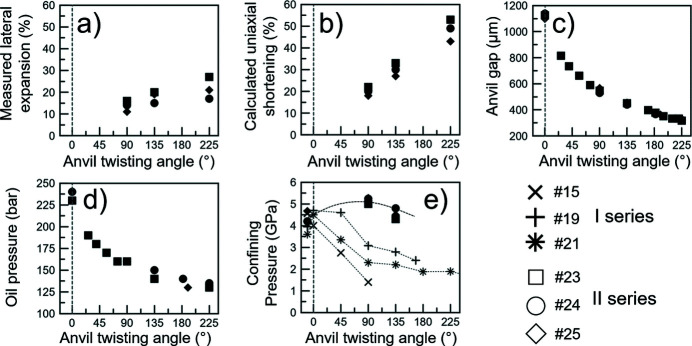
Lateral expansion (*a*), sample uniaxial strain (*b*), anvil gap (*c*), oil pressure (*d*) and confining pressure in the cell/samples (*e*) with anvil twisting angle. In each diagram, the dashed line (0° twisting) defines the beginning of deformation. (*e*) Comparison of confining pressure (GPa) between runs of the second series of experiments (#23, #24, #25) and the other three (#15, #19, #21) from the first series (Table 2 ▸). For #23, #24, #25, the dashed line is a guide-to-the-eye for the confining pressure trend.

Fig. 6 ▸ shows the lateral expansion measured on the sample (µm), calculated uniaxial shortening (µm), measured anvil gap (µm), apparatus oil pressure (bar), and trends of confining pressure (GPa). Here, the experiments were stopped when an anvil gap of ∼300 µm was reached, in order to keep a field of view [H, Fig. 5 ▸(*b*)] suitable for XRT and microstructural interpretations.

The lateral expansion [Fig. 6 ▸(*a*)] and uniaxial shortening [Fig. 6 ▸(*b*)] increase with increasing twisting angles. The maximum shortening is ∼55% and the maximum lateral expansion is ∼30% at the twisting angle of 225° for the same sample [Figs. 6 ▸(*a*) and 6(*b*)]. The amount of uniaxial shortening is therefore not fully reflected in the lateral expansion.

The anvil gap [Fig. 6 ▸(*c*)] and apparatus oil pressure [Fig. 6 ▸(*d*)] decrease with increasing twisting angle. The decrease of the anvil gap is more pronounced at the beginning of the twisting. The apparatus oil pressure follows a similar trend as the gap reduction [Figs. 6 ▸(*c*) and 6(*d*)]. The observed loss of the apparatus oil pressure [Fig. 6 ▸(*d*)] could suggest that the confining pressure transferred to the sample decreases as torsion is performed, and could prompt users to regulate the oil pressure to maintain the hydro­static pressure inside the assembly. However, this can lead to an early experiment termination if the anvil gap reduces further.

A comparison in hydro­static pressure (in GPa) is given [Fig. 6 ▸(*e*)] between runs from the second series of experiments (#23, #24, #25) and three other runs (#15, #19, #21) from the first series.

Runs #23, #24 and #25 show an increase in confining pressure of the order of ∼1 GPa from the start of the experiments to 90° anvil twisting [Fig. 6 ▸(*e*)], when most of the loss in oil pressure (bar) occurs [Fig. 6 ▸(*d*)]. Then, at higher twisting angles, the three runs show a decrease in pressure of ∼0.8–1 GPa. Conversely, the runs #15, #19 and #21 show a decrease in confining pressure of ≥2 GPa from the start to the end of the experiments [Fig. 6 ▸(*e*)]. This first series was conducted with tomographic datasets acquired under HT, whereas in the second series (#23, #24, #25) the tomographic datasets were acquired under room temperature. This suggests that a longer exposure of the assembly to HT causes the pressure-transmitting medium [gasket, Fig. 1 ▸(*a*)] to become less effective, and a considerable loss of confining pressure occurs.

## 5. XRT post-processing

The post-processing workflow (after reconstruction) of the tomography images consists of: selection of a representative volume (RV) in the whole sample image; application of various filters; segmentation and binarization; post-segmentation processing; quantitative analysis.

A detailed description of each of these steps is given in the following sections. The first steps determine the quality of the subsequent quantitative analysis.

**Figure 7:**
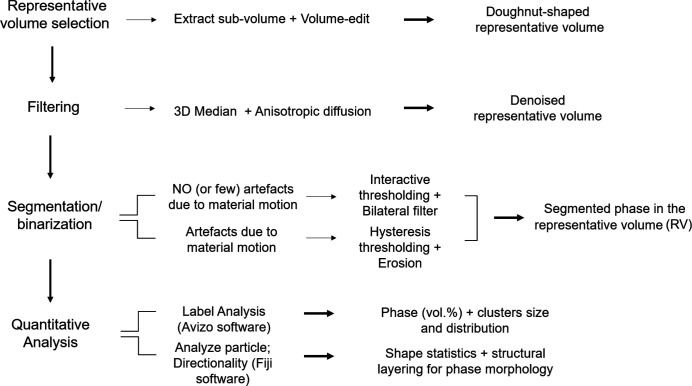
Workflow overview of the XRT post-processing and analysis.

All steps before the analysis were performed using the commercial software *Avizo* (https://www.thermofisher.com/fr/fr/home/electron-microscopy/products/software-em-3d-vis/avizo-software.html). The quantitative analyses on XRT images were performed with both *Avizo* and *Fiji*. The latter is a specific distribution of the open-source program *ImageJ* (https://imagej.nih.gov/ij/). Fig. 7 ▸ shows an overview of the workflow on post-processing and analysis.

### 5.1. The representative volume

The investigated volume should be as representative of the sample geometry as possible to avoid biases. When the aim is to obtain statistical information, a large volume is required to obtain a robust final analysis.

**Figure 8:**
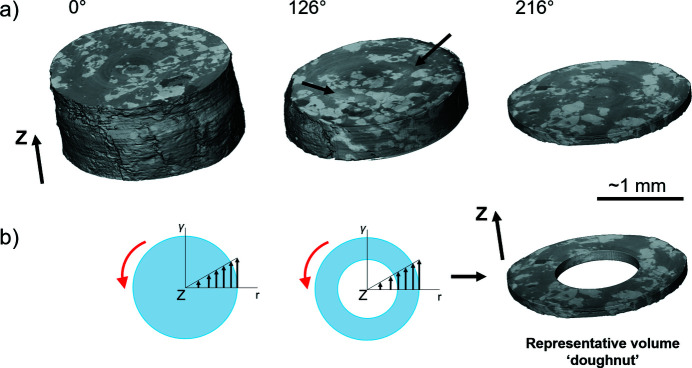
XRT render of sample #17 (Table 2 ▸) with increasing twisting angle. *Z* is the torsion axis. (*a*) Representative example of absorption-contrast XRT between different phases (from brightest to darkest: garnet, secondary mineral; pyroxene, matrix mineral; quartz, accessory mineral). XRT renders gradually decrease in height with twisting angle due to anvil gap reduction. Arrows (126° twisting) show rings and shading artefacts. (*b*) Theoretical transfer of the strain in a solid cylinder and in the representative volume. γ is strain and *r* is radius. Red arrows show the direction of torsion. Sequential black arrows show the strain gradient along the radius.

The RV here is further constrained by two experimental limitations: (1) the presence and distribution of the artefacts (see Section 2.4), and (2) shorter image (height) of the sample at higher angles of twist. For limitation (1), the artefacts are usually more pronounced in the inner and outer regions of each reconstructed image. The artefacts remain difficult to completely remove at this stage even with filtering or denoising modules. Therefore, we choose not to consider these areas for the RV, and crop them out. The other limitation (2) is due to the gap between the outer edges of the anvils, which shortens during the torsion [Fig. 6 ▸(*c*)]. This makes the window for XRT smaller [Fig. 5 ▸(*b*)], leading to a reduced volume size of the tomography [Fig. 8 ▸(*a*)]. For the purpose of observation and statistics consistencies, for each sample we select similar sizes of RVs for all tomographic datasets (*i.e.* at different twisting step). The size of the RV for each sample is chosen on the basis of the height of the last image at the highest twisting angle.

The ‘Extract sub-volume’ and the ‘Volume-edit’ tools in *Avizo* are used to obtain the RV. ‘Extract sub-volume’ is used to define the bounding box containing the actual sample and remove portions of the tomography image including parts of the assembly or the anvils. ‘Volume-edit’ is used to apply a user-defined cropping, based on a geometric 3D mesh. Using a cylindrical mesh, two croppings are made at two different radii to remove the outer and inner regions of the sample, *i.e.* the two regions where the artefacts are more pronounced [Fig. 4 ▸(*b*)]. The resulting cropped volume obtained at this stage is a hollow cylinder. This cylinder is then cropped along the height, leading to a doughnut-shaped volume [Fig. 8 ▸(*b*)] with boxes of 1600 × 1600 × 150 voxels on average.

The representative doughnut-shaped volume can allow a microstructure investigation which is consistent with the transport of matter in the torsion geometry [Figs. 5 ▸(*b*) and 8(*b*)]. Moreover, the strain difference between the outer and inner walls of the hollow cylinder is smaller than for the whole cylinder [Fig. 8 ▸(*b*)].

For detailed observations of the 3D microstructures at smaller scale in selected regions within these ‘doughnuts’, an extraction of volume boxes (150 × 150 × 100 voxels) is performed using the ‘Extract sub-volume’ tool.

### 5.2. Filtering and segmentation

Noise-reduction and edge-preserving filters are used to erase, or reduce, some of the remaining artefacts in the RVs. The basic ‘3D Median’ filter is often used in this study. We run the ‘Anisotropic diffusion’ module to smooth rings artefacts if still present.

For the grey-levels images, choices made by the user for filters parameters can be non-unique. This results in uncertainties on the quantitative analysis performed on the segmented RV. Here, the influence of these choices was estimated by manually iterating over a range of filter parameter values, from visual inspection of the filtered image. Then, an uncertainty range was obtained above and below which the filtering was considered incorrect. This uncertainty range was subsequently taken into account to estimate the total uncertainties in a 3D quantification for our RV tomographic datasets (see Section 6.2).

**Figure 9:**
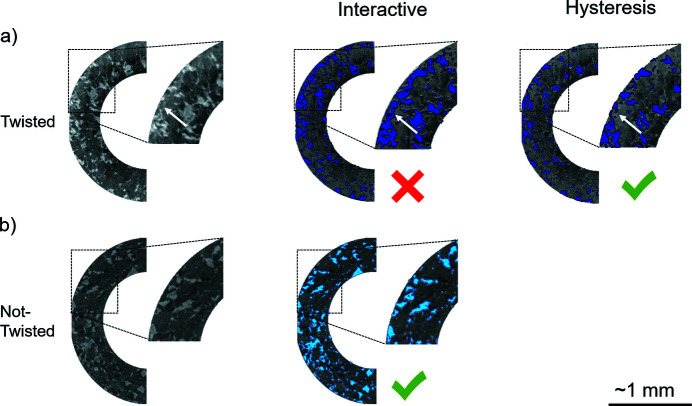
Comparison between Interactive and Hysteresis thresholding in an aggregate of olivine + serpentine (negative contrast: serpentine is brighter; olivine is darker). (*a*) Tomography after twisting. Sample motions artefacts are present, and Hysteresis is used. White arrows show local ‘unwanted’ segmentation (see text) that is present if Interactive is used. The ‘unwanted’ segmentation is not present if Hysteresis is used. (*b*) Tomography before twisting. Artefacts are not observed, and Interactive is used.

After filtering, the doughnut images were transformed into binary by applying image segmentation through thresholding tools, assigning specific grey-level ranges to each phase. In this study, we mainly used the Interactive and Hysteresis thresholding available in *Avizo*. Fig. 9 ▸ shows a comparison between these thresholding tools in the reconstructed images.

**Figure 10:**
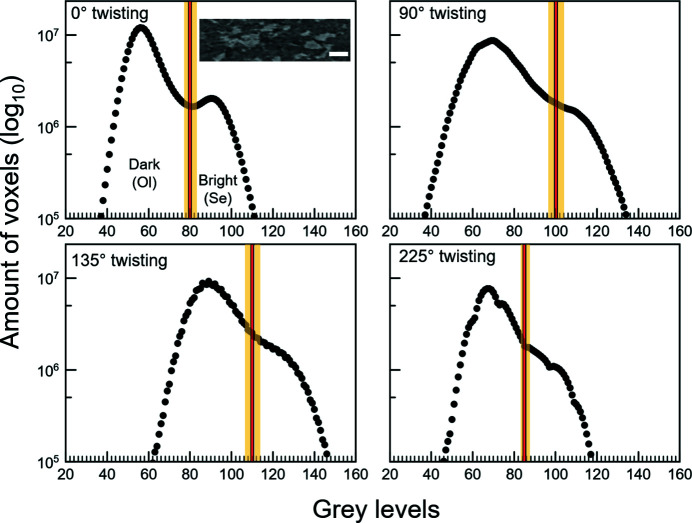
Example of histograms (run #18) of image grey-levels (negative contrast) at different anvil twisting angles showing selected thresholds (red) with uncertainties (yellow) to segment the brighter phase (Se, serpentine). Ol is olivine. The upper-left quadrant inset shows a representative image of the sample; the white bar corresponds to ∼100 µm.

The Interactive tool prompts the user to set the grey-level intervals manually with visual feedback. However, in some cases this tool segments noise, *i.e.* what we consider ‘unwanted’ areas for segmentation. Such areas do not correspond to the phase locations in the grey-level reference image [Fig. 9 ▸(*a*)]. In this case, one solution is to use a post-segmentation filter, such as a bilateral filter. Alternatively, we used the Hysteresis thresholding, where a grey-level threshold is selected above which the segmentation will be applied. This implies that this tool works well when the mineralogical phase of interest has the highest grey levels in the image. When the phase of interest has the lowest intensities, a grey-scale inversion (negative contrast) is necessary. Fig. 10 ▸ shows a representative example of a selection of thresholding values for both Interactive and Hysteresis.

Segmentation uncertainties are also estimated with the same strategy as for filtering uncertainties. These estimations are based on the user’s choices of thresholding parameters to segment the contouring of the phase of interest from visual inspection. Uncertainty ranges on image grey levels (Fig. 10 ▸) were therefore obtained above and below which segmentation was considered incorrect. The visually estimated thresholds and uncertainty ranges fall where there is a change of slope on the grey-levels histograms (Fig. 10 ▸). The change of the slope indicates the boundary between the different phases.

The segmentation uncertainty ranges obtained from visual inspection (Fig. 10 ▸) were then taken into account for estimating the total quantifications uncertainties during quantitative analyses performed on the segmented RV (see Section 6.2).

### 5.3. Post-segmentation

After thresholding, to further improve the segmentation to be as close as possible to the actual contouring of the phase of interest, the ‘Erosion’ tool was used when necessary and often where Hysteresis thresholding was previously used. This is because the Hysteresis thresholding does not always preserve the edges of the phase. Erosion is one of the manual correction tools in *Avizo* that offers the possibility to correct and ‘clean’ automated segmentation (*e.g.* Zhu *et al.*, 2011 ▸), possibly revealing morphological details previously hidden in the sample image (*e.g.* Liu & Regenauer-Lieb, 2021 ▸).

Finally, whether the applied segmentation would or would not give satisfactory results was firstly judged on the basis of visual feedback. Here, the phases respective volumes were known beforehand, and it is safe to assume that no chemical reactions and nucleation of new phases occurred during the experiments (*i.e.* temperature well within the stability field of minerals investigated, low temperature). The calculated total volume percentage of the segmented phase was therefore used as further confirmation of the satisfactory segmentation.

The analyses are then run on the ‘clusters’ of the segmented phase. The term ‘cluster’ indicates a group of pixels (if in 2D) or voxels (if in 3D) in the segmented (binary) images that belong to the same phase and are connected. Each cluster is an individual particle or a structure of the segmented phase with its own morphology and size, observed and identified in 2D or 3D. The clusters are arbitrarily defined by the selected voxel range. Table 4 ▸ summarizes our arbitrary classification of the cluster size, as cluster area in pixels or micrometres for 2D investigation, and number of voxels in the cluster for the 3D investigation. Here a pixel is 1.3 µm × 1.3 µm; a voxel is 1.3 µm × 1.3 µm × 1.3 µm. For comparison, the volume of the RV is of the order of 10^8^ voxels.

## 6. Deformation microstructures

### 6.1. Analysis tools and quantifications

The first part of the analysis is performed on *Avizo* and run on the 3D doughnut-shaped RV to (i) obtain phase volume proportions, (ii) observe the morphology of the clusters of a phase, and (iii) obtain the degree of connectivity of the clusters of a phase (see Section 6.2) with increasing deformation.

The second part of the analysis is performed on *Fiji*, and run on selected 2D unrolled sections extracted from the RV at a selected radius using a Matlab script developed by M. Thielmann (personal communication) from the University of Bayreuth (BGI, Germany). The extracted unrolled sections can allow 2D microstructural analysis consistent with the transport of matter in torsion [Figs. 5 ▸(*b*) and 8(*b*)], along the whole RV perimeter.

First, the 2D analysis was used to obtain statistics on shape descriptors of the clusters. The shape descriptors (https://imagej.nih.gov/ij/docs/menus/analyze.html) used here are:

(i) Area (µm^2^).

(ii) Aspect ratio – the ratio of the major axis over the minor axis of the cluster-fitting ellipse. It gives information on shape anisotropy.

(iii) Circularity – defined by the formula 4π(area/perimeter^2^). The perimeter corresponds to the length of the contouring of the clusters. The circularity can range from values of 0.1 to 1, where 1 represents a perfect circular shape. In comparison with the aspect ratio, the circularity takes into account the complexity of the contouring of the clusters.

2D simplification affects the measured lengths on a 2D section relative to the actual lengths in 3D: the selected sections unlikely cross the features on their longest dimension, for instance. This impacts the measured areas, which here are a lower bound for the actual features. The aspect ratio and circularity should be less or not affected since they are a ratio, if the bias is similar for all measured lengths.

Then, 2D analysis was used to obtain the orientation in angle (°) of clusters’ boundaries with respect to the shear direction. This was done through the ‘Directionality’ tool in *Fiji* (https://imagej.net/plugins/directionality). The directionality computes the distribution of the orientation (from −90° to 90°) of the clusters’ boundaries with respect to the horizontal axis of the image (here, the shear direction) (*e.g.* Liu, 1991 ▸). It gives orientations of inter-phase boundaries, between the secondary (less abundant phase) and the phase in the matrix. The distribution of the angles here may be biased if the streamlines for the transport of matter do not follow a circular geometry.

### 6.2. Results

Here, we give representative examples of deformed microstructures and quantifications on the aggregates of olivine + serpentine. The results focus on the secondary phase in the aggregates, *i.e.* the serpentine. The process was refined on the whole series of experiments, and the results shown here are extracted from run #18 analysis.

#### 6.2.1. 3D

**Figure 11:**
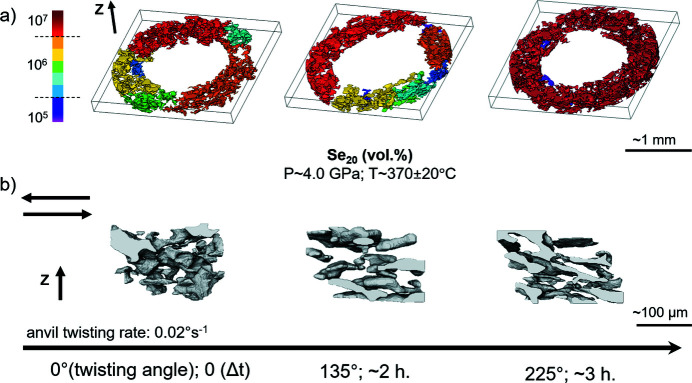
Renders of sample #18 (Table 2 ▸) showing the serpentine morphology with increasing anvil twisting angle. *Z* is the torsion axis, *P* is pressure, *T* is temperature, Se is serpentine, the black arrow at the bottom indicates increasing anvil twisting angle, where Δ*t* corresponds to the twisting duration. (*a*) Clusters in the RV. The bar shows the voxel amount in the clusters. The bounding boxes are ∼1600 × 1600 × 150 voxels. (*b*) Clusters in smaller regions of interest within the RV. The bounding boxes are ∼150 × 150 × 100 voxels.

The 3D microstructure of serpentine shows the size of the largest cluster increasing with increasing twisting [Fig. 11 ▸(*a*)]. At smaller scale [Fig. 11 ▸(*b*)], it is possible to observe the morphology of the clusters changing with increasing deformation. From a random distribution, the clusters orient parallel or subparallel to the shear direction [Fig. 11 ▸(*b*)]. At the last stage of deformation (225° twisting), their morphology reflects the sense of transferred shear [Fig. 11 ▸(*b*)].

**Figure 12:**
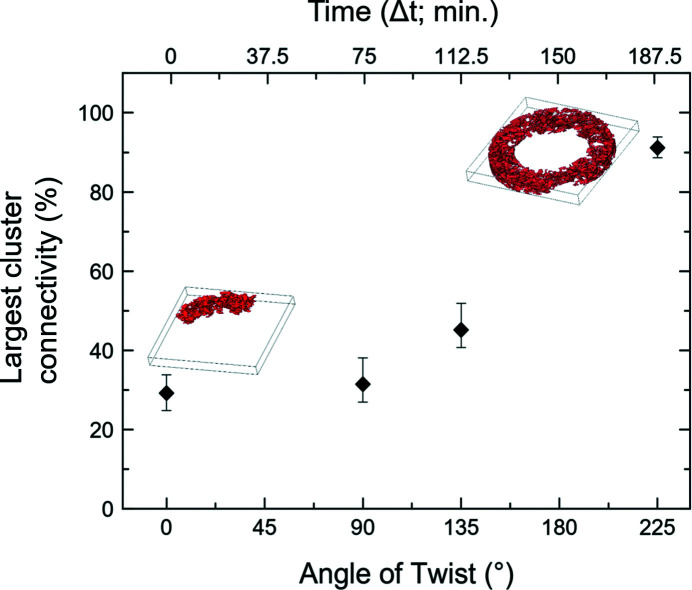
Evolution of connectivity of the largest cluster [Fig. 11 ▸(*a*)] with increasing anvil twisting angle (angle of twist) and twisting duration (time). Error bars show the total connectivity uncertainties (Table 5 ▸).

We estimate here the degree of connectivity of the serpentine in the aggregate. Following Kaercher *et al.* (2016 ▸), this can be done by taking into account the size of the largest cluster. The connectivity is calculated by dividing the volume of the largest cluster by the total volume of the phase present in the RV [after Kaercher *et al.* (2016 ▸)]. Fig. 12 ▸ shows the evolution of the largest cluster connectivity with increasing deformation.

At first, the connectivity of serpentine seems constant from 0° to 90° anvil twisting angle, with values of ∼30%. Above 90° twist, the trend becomes steeper with values increasing from ∼30% to ∼90% of connectivity at 225° anvil twisting angle (Fig. 12 ▸). The uncertainties on connectivity (Table 5 ▸) are evaluated taking into account the uncertainties ranges estimated for image segmentation and filtering. These uncertainty ranges influence the result of the segmented phase volumes, hence the connectivity calculation.

#### 6.2.2. 2D

**Figure 13:**
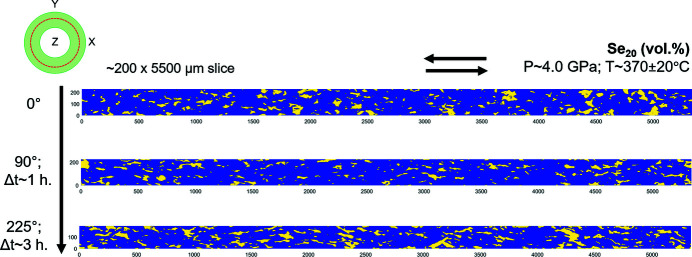
2D serpentine clusters (yellow) with increasing anvil twisting (°) in unrolled sections extracted from RVs of run #18 (Fig. 11 ▸). The upper-left inset shows the approximate location from where the unrolled sections are extracted (red line). *Z* is the torsion axis, *P* is pressure, *T* is temperature, Se is serpentine. The arrows at the top show the sense of shear. Number labels of unrolled sections are approximate dimensions in micrometres. Δ*t* corresponds to the twisting duration.

Fig. 13 ▸ shows the 2D evolving microstructure of the serpentine in the unrolled sections. The serpentine clusters become elongated and sub-parallel to the shear direction at 90° anvil twisting (Fig. 13 ▸). At 225° anvil twisting, the clusters increase in size, rotate and display the sense of the transferred shear (Fig. 13 ▸). This is consistent with the observations at smaller scale in 3D [Fig. 11 ▸(*b*)].

**Figure 14:**
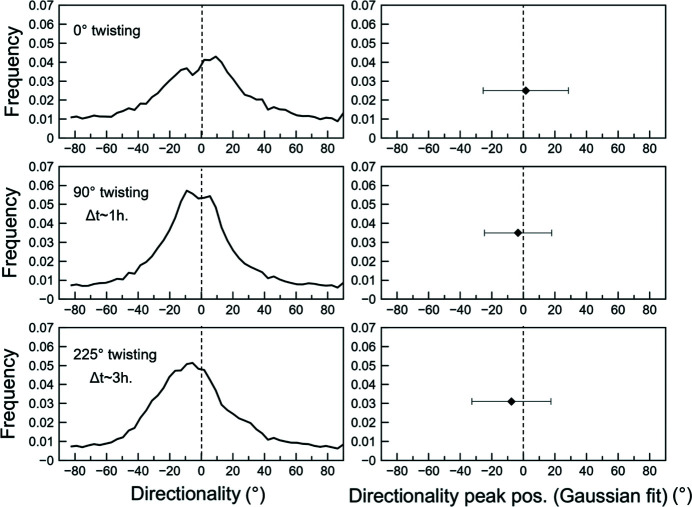
Directionality distributions with increasing anvil twisting (°) showing clusters’ (Fig. 13 ▸) boundaries orientations with respect to the shear direction. Δ*t* corresponds to the twisting duration. Panels on the right show the peak position and HWHM (half-width-half-maxima, error bars) from Gaussian fits performed on the directionality distributions.

The directionality (see Section 6.1) shows the orientations of serpentine clusters’ boundaries (interphase) with respect to the shear direction at different twisting (Fig. 14 ▸). The number of cluster boundaries oriented parallel to the shear direction increases between 0° to 90° twist (Fig. 14 ▸). Beyond 90° twist, the overall directionality distribution skews towards negative angles of ∼10–20° (Fig. 14 ▸). These changes in orientations and distributions of interphase boundaries with shear can give information on morphological anisotropy for both phases, with respect to the shear direction.

**Figure 15:**
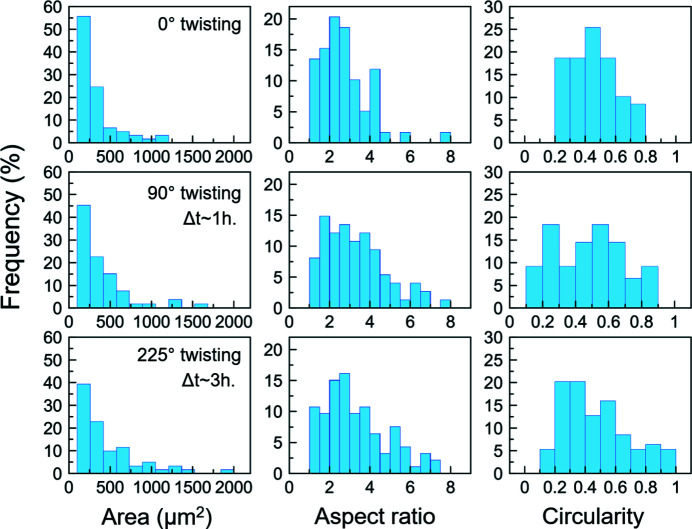
Histograms showing area, aspect ratio and circularity of the clusters (Fig. 13 ▸) with increasing anvil twisting angle (°). The aspect ratio is defined as the ratio major-axis/minor-axis of the fitting ellipse to a single cluster structure. Circularity is defined as 4π(area/perimeter^2^) of a single structure.

The statistics on shape descriptors (area, aspect ratio, circularity) are presented in Fig. 15 ▸. They show that the population of smaller serpentine clusters decreases with increasing twisting angle. The aspect ratio increases from most values below 4, up to 8 with deformation. The aspect ratio also shows an increase in frequency of lower aspect ratios at the last stage of deformation (around 2–3; Fig. 15 ▸).

The circularity takes into account the actual contouring of the clusters rather than a fitting ellipse to a given structure, and better characterizes the morphological anisotropy in the clusters rather than the aspect ratio. This make it more suitable to characterize complex structures such as those of the clusters at the last stage of deformation (Fig. 13 ▸). The circularity distribution is skewed towards values close to zero at 225° anvil twisting (Fig. 15 ▸). This indicates an increasing population of serpentine structures deviating from a morphology of a sphere and becoming complex at the last stage of deformation (Fig. 13 ▸).

One observation that can be made here is that the aspect ratio is obtained from fitting an ellipse to a given structure, and does not capture the actual complexity of the structures developing between 90 and 225° of twist. The increase in 3D connectivity, seen in the previous section, is a consequence of these structures connecting, and becoming more complex structures. Thus, information such as circularity that takes into account the complexity of shapes should be more meaningful than aspect ratio, for the latest stages.

## 7. Discussion

Here, we summarize our observations on the coupling between pressure and deformation in the RoToPEc, and possible improvements for deformation experiments under high pressure and high temperature coupled to synchrotron XRT.

### 7.1. Pressure and anvil gap

We examined how the anvil gap, apparatus oil pressure, sample lateral expansion and shortening evolve with increasing deformation in the press (Fig. 6 ▸). Without a feedback controlling device, the lateral extrusion of the assembly induces a loss of apparatus oil pressure. It does not necessarily correspond to a loss of pressure in our samples in runs from the second series of experiments [Fig. 6 ▸(*e*)]. Therefore, any future work on feedback mechanisms in order to better control the hydro­static pressure on the samples should rely on pressure measured *in situ* when available, rather than load.

The decrease of the anvil gap [Fig. 6 ▸(*c*)] limits the window for the X-ray tomography analysis [Figs. 5 ▸(*b*) and 8 ▸(*a*)]. The decreasing gap can ultimately result in anvils contact for large twists/high-temperature experiments. This limits the duration of the deformation experiment, and possibly leads to blow-out and anvil failure. As of now, a compromise has to be found between achieving large shear strains and keeping a sufficiently large height of view for the microstructural investigation in the tomographic datasets. In the series of experiments where the tomographic datasets were acquired on quenched samples, the gaskets kept a larger height of view than those where the whole experiment was under HT. This suggests that the pressure-transmitting medium (gasket) loses performance over time under elevated temperature. In other words, lateral extrusion is enhanced by longer exposure of the assembly to HT. Quenching the assembly after each step of twist is, as of now, the most efficient way to reduce the extrusion and loss of pressure in the cell/sample.

This process leads to a complex pressure–temperature–stress history on the sample, which is unsatisfactory. Future work on the design of the assemblies and pressure-transmitting medium is therefore required for making progress. Improvements may also concern new anvil materials or designs, for a better efficiency in pressure generation, and/or for X-ray transparency that would allow a higher field of view.

### 7.2. Efficiency and use of the RoToPEc for shear deformation experiments

The deformation field in the sample is a result of combined uniaxial shortening/lateral expansion [Figs. 6 ▸(*a*), 6(*b*)], and torsion. The microstructure can be affected in multiple locations or in the whole volume of the sample, which makes the interpretation of deformation behaviour complex.

An increase in pressure for the second series of experiments is occurring during the first step of twisting (90°) [Fig. 6 ▸(*e*)]. The measured sample lateral expansion does not fully compensate the calculated uniaxial shortening [Fig. 6 ▸(*a*), 6(*b*)]. This is consistent with the sample gaining pressure rather than experiencing shear at this stage, *i.e.* at ≤90° anvil twist. At this twist condition, the simple shear strains transferred to the samples are low (Table 3 ▸), with the microstructures mostly showing cluster elongation (flattening, Figs. 13 ▸, 14 ▸). This suggests pure shear is transferred to the sample at the beginning, rather than simple shear. Rotation in the microstructure, reflecting the transferred sense of shear [*e.g.* Figs. 11 ▸(*b*), 13 ▸], occurs after 90° twist and indicates an effective transfer of simple shear. Therefore, a main observation of this work is that, after an initial stage of pressurization and increase of friction in the assembly parts, the coupling between the rotating anvil inducing torsion and the sample becomes really effective at twisting angles ≥90°. Although the strain rates are different, this value seems consistent with the low strains found by Berg *et al.* (2017 ▸), on samples recovered from twist experiments in a rotational PEc (roPEc).

Future work should focus on employing hollow samples to simplify the transport field of matter, as done in lower pressure experiments (*e.g.* Dobson *et al.*, 2020 ▸). This would minimize the potential biases when using 2D sections outlined in Section 6.1, and allow an easier use of the 3D information. It would also simplify fractures patterns, which follow in 3D a helicoidal distribution within our samples.

In order to fully characterize the deformation behaviour of materials, stress measurement is required. Use of the RoToPEc to collect tomographic datasets and perform global or local stress measurements using X-ray diffraction, either in angular-dispersive mode with a monochromatic beam or in energy-dispersive mode with multiple Ge-detectors, could be a mid- to long-term goal. This would require modifications of anvil design and material.

### 7.3. XRT data perspectives

One of the major difficulties during our reconstructions and post-processing was the presence of persistent motion artefacts. These are due to the local physical motion in the sample caused by deformation. These motion artefacts are difficult to completely avoid during acquisition, and erasing them during either reconstruction or post-processing is equally difficult. They can hamper the proper investigation on the microstructure, influence the errors on the volume quantifications, connectivity and shape descriptors of the phases.

In order to avoid these artefacts, we let the sample stabilize after each deformation step (*i.e.* anvil twisting angle) before acquiring the X-ray tomography, for at least ∼30 min. A much more attractive solution would be to carry out the tomography fast enough so that motion in the samples during the acquisition is small. *In situ* X-ray fast tomography at HT was reported for the 2BM beamline at the APS synchrotron by Xiao *et al.* (2012 ▸). They conducted annealing experiments to observe phase transition or dehydration reactions in rocks at ambient pressure and temperature up to ∼900 K, and acquired *in situ* fast tomography down to the order of 200 ms for each scan. At the TOMCAT beamline at Swiss Light Source, experiments under temperature or with low-pressure deformation devices can be carried out with acquisitions at second or even sub-second time scales (Marone *et al.*, 2020 ▸; Maire *et al.*, 2016 ▸). At the high-pressure PSICHE beamline (Synchrotron SOLEIL), *in situ* X-ray fast tomography (of the order of less than a second for a complete tomogram) is possible with the UToPEc apparatus (*e.g.* Boulard *et al.*, 2018 ▸; King *et al.*, 2019 ▸; Giovenco *et al.*, 2021 ▸). This variation of the PEc design allows high-pressure (>1 GPa), high-temperature experiments but does not have deformation capacity. The RoToPec is the only variation that allows deformation and tomography. However, the current type of motors for the anvil rotation imposes a duration of ∼20 min per acquisition. Implementing a faster tomographic dataset acquisition requires heavy hardware modifications that should be the scope of future developments. Such developments would be highly desirable to minimize the motion in the samples, reduce the amount of work during pre-processing or post-processing, and improve *in situ* imaging of deformation microstructures under HP/HT.

Such technological breakthroughs would for instance allow study of the microstructural evolution of rocks relevant for the earth’s lithosphere, upper mantle and subduction zones (down to ∼150–200 km depths), which are of paramount importance for understanding the upper part of earth convection and deep seismicity. Deformation investigation at higher temperatures and pressure (intermediate to lower mantle) are limited by the extrusion extent of the assembly/sample that is occurring during torsion and enhanced at high temperatures.

A final point is on the *in situ* absorption-contrast XRT used, which best captures heterogeneity and morphological features relative to the phases in the aggregates. As of now, it is not very adequate for capturing features such as porosity, cracks or fractures. The grey levels of pores or fractures would not be easily distinguished from the grey levels of the phases in the images. In this case, *in situ* phase-contrast X-ray tomography may be an alternative to capture porosity, cracks or fractures, and may become an important tool to work on continental and oceanic crust related processes in the earth.

## 8. Conclusions

We explored the use of the RoToPEc to perform torsion under HP/HT and collected *in situ* X-ray tomography on deforming multi-phase aggregates. We were able to observe the evolution of the microstructures with strain on a representative volume consistent with the transport of matter in torsion, providing examples of 2D and 3D quantifications such as phase clusters connectivity, orientations of interphase boundaries with shear direction, clusters aspect ratio and circularity. Excluding spatial resolution, which was beyond the scope of this study, the main RoToPEc limitations remain from the hardware point of view: the speed of tomography acquisition allowed by the motors, and the height of the field of view for imaging. The RoToPEc is suitable for transferring high shear strains, representative of large shear strain environments at conditions of earth crust to uppermost mantle. This tool has a strong potential to shed new light on the study of polyphase aggregates and rocks under high pressures and temperatures, in particular to understand the distributions of strain, stresses and strain localization processes. The conditions investigated are a starting point for broadening the RoToPEc pressure and temperature and time resolution conditions for quantitative deformation experiments. Pushing these boundaries will require technological developments such as new designs for high-pressure cells and anvils in the RoToPEc, or adaptation of devices compatible with fast (s timescale) tomography setups such as the UToPEc, for deformation studies.

## Figures and Tables

**Table 1:** Linear relation between power [W] and temperature [°C] used to fit trends in Figs. 3 ▸(*a*), 3(*b*) *T* is temperature, *P* is power, Res. std dev. is residual standard deviation and *R* ^2^ defines the goodness of the fit.

		Fit: *T*[°C] = *a* *P*[W] + *b*			
Load (bar)	Pressure (GPa)	*a*	*b*	Res. std dev.	*R* ^2^
250	∼4	1.981	29.901	14.657	0.994
150	∼2	1.963	32.551	14.581	0.995

**Table 2:** Runs conducted in the RoToPEc Starting materials are powders mixtures (particle size: 50–100 µm), except for run #17 where a core-drilled sample was used (see text). ‘*Ex situ*’: experiments performed at IMPMC. Se: serpentine, Ol: olivine, Grt: garnet, Omp: pyroxene.

		Pressure (GPa)				
Run (#)	Composition (phase, vol%)	Maximum	Minimum	Temperature (°C)	Maximum angle of anvil twist	Use of run
15	Se10Ol90	∼4	1.4	330	90	Pressure (GPa) and anvil gap
	Se: 9.9 ± 0.5†					
15b	Se10Ol90	∼4		∼330	225	*Ex situ* run, strain estimates
	Se: 10.4†					
17	Grt35Omp60	∼3	∼1	430	216	Representative volume description‡
18	Se20Ol80	∼4	∼3	370	225	Microstructure quantifications
	Se: 16.6 ± 0.9†					
18b	Se20Ol80	∼4		∼370	No twist	*Ex situ* run, strain estimates
19	Grt15Omp85	4.7	2.4	500	168	Pressure (GPa) and anvil gap
21	Grt70Omp30	4.5	1.6	500	270	Pressure (GPa) and anvil gap
23§	Se10Ol90	5.2	4.2	370	225	Strain calculations, anvil gap and load
	Se: 9.6 ± 1.6†					
24§	Se20Ol80	5.3	4.5	330	225	Strain calculations, anvil gap and load
	Se: 18.9 ± 2.5†					
25§	Se10Ol90	∼5	∼4	430	225	Strain calculations, anvil gap and load
	Se: 10.6 ± 1.3†					

† Calculated phase volume in tomographic datasets during post-processing (see Section 5).

‡ See Section 5.

§ Second series of experiments, where tomographic datasets were always acquired at room temperature, after quenching the samples after each twisting step (see Section 2.4).

**Table 3:** Strain measurements Se: serpentine. Ol: olivine. ɛ: uniaxial strain. γ: shear strain. ɛ_E_: equivalent strain. Entries with two values separate by ‘/’ indicate calculations from markers close to the sample edge and centre, respectively.

		Pressure (GPa)			Twisting angle (°)		Strains			Total strain rates (s^−1^)		
Run (#)	Composition (vol.%)	Max	Min	Temperature (°C)	Anvil	Sample (θ)	ɛ†	γ	ɛ_E_ (%)‡	ɛ	γ	ɛ_E_ ‡
23	Se10Ol90	5.2	4.2	370	0							
					90	47	0.2	1.5	177.1			
					135	59	0.3	2.2	255.5			
					225	102	0.5	5.1	592.6	3.9 × 10^−5^	4.7 × 10^−4^	5.5 × 10^−4^
24	Se20Ol80	5.3	4.5	330	0							
					90	45	0.2	1.2 / 1.1	144.7 / 132.1			
					135	60	0.3	1.9 / 1.5	219.0 / 174.1			
					225	103	0.4	4.1 / 2.9	479.5 / 334.3	3.9 × 10^−5^	3.8 × 10^−4^ / 2.7 × 10^−4^	4.4 × 10^−4^ / 3.1 × 10^−4^
25	Se10Ol90	∼5	∼4	430	0							
					90	61	0.2	1.4	160.8			
					135	74	0.3	2.0	228.3			
					225	118	0.5	4.5	516.8	4.9 × 10^−5^	4.1 × 10^−4^	4.8 × 10^−4^

† Values at 90° and 135° anvil twists are estimated from total uniaxial strain rate, values at 225° anvil twist are calculated from recovered samples (see text).

‡ Calculated using equations (5).

**Table 4:** Classification of cluster size for 2D and 3D images (arbitrarily defined)

	Clusters 2D				Clusters 3D		
Units	Small	Medium	Large	Units	Small	Medium	Large
µm^2^	50 ≤ *x* < 100	100 ≤ *x* < 1000	*x* ≥ 1000	voxels	10^1^ < *x* ≤ 10^3^	10^4^ ≤ *x* ≤ 10^5^	10^6^ ≤ *x* ≤ 10^7^
pixel^2^	∼30 ≤ *x* < ∼60	∼60 ≤ *x* < ∼590	*x* ≥ ∼590				

**Table 5:** Uncertainties estimation of the largest cluster connectivity (%) All the uncertainties (+ and −) refer to connectivity (%). The total connectivity (%) uncertainties corresponds to the error bars in Fig. 12 ▸. RV: representative volume. Se: serpentine.

					Connectivity (%) uncertainties					
					Thresholding				Total connectivity (%) uncertainties	
Anvil twist angle (°)	RV (mm^3^)†	Se volume (mm^3^)†	Se (vol%)†	Largest cluster connectivity (%)	Tool‡	+	−	Filters§ (+, −)	+	−
0	0.462	0.071	15.433	29.197	Interactive	3.193	2.994	1.381	4.574	4.374
90	0.396	0.066	16.740	31.450	Hysteresis	5.292	3.113	1.381	6.672	4.494
135	0.442	0.077	17.437	45.161	Hysteresis	5.292	3.113	1.381	6.672	4.494
225	0.382	0.065	16.954	91.152	Interactive	1.313	1.11	1.381	2.694	2.494

Average	0.421	0.070	16.641							
Standard deviation	0.038	0.006	0.856							

† Calculated using the software *Avizo*.

‡ Segmentation tool used in *Avizo*.

§ Refers to ‘Median’ and ‘Anisotropic diffusion’ filters (see text).
